# Dr Douglas Gawn

**Published:** 1984-10

**Authors:** 


### Obituary

### Douglas Gawn

Douglas Gawn, doyen of general practitioners in South Bristol, died in Southmead Hospital on 9th August 1984, aged 70. He had been in practice in Knowle for 38 years following his father who had practised from the same house for 40 years before him.

Harold Douglas Townshend Gawn was born on 7th September 1913 above the surgery, delivered by his father. He was educated at Clifton College and graduated from Bristol University in 1938. After a short spell at the General Hospital he joined his father in general practice.

When war broke out he enlisted in the Royal Army Medical Corps and was posted to the Far East, arriving in Singapore just ten days before it fell to the Japanese. The remainder of his war was spent in Changi Goal and in the harshness of prison camps in Burma and Thailand. On his returning to Bristol he succeeded his father in 1946. He retired from NHS practice in 1976 but continued with private work until a month before his death.

Many will remember him best for his work with the St John Ambulance Brigade. He joined the Totterdown Division as the Divisional Surgeon in 1947, being later promoted to Corps Surgeon. With the formation of the County of Avon in 1974 he was appointed the first Area Surgeon for Bristol and North Avon, a post he held until January 1984. He was honoured as a Serving Brother of the Order of St John in 1967, being promoted to Officer Brother in 1976 and to Commander this year.

President of the local Scout Association, a regular churchgoer, he was a Christian who practised his beliefs in spite of his horrific wartime experiences.

His love of poetry and of the country-side, his love of his family and, old-fashioned as he was, his love of his patients were his guiding lights. He will be sorely missed by all his colleagues as 'the little man with the great heart'.

At his funeral service the Church of St Barnabas, Knowle West was full to over-flowing; the St John Ambulance Brigade formed a Guard of Honour. The service was conducted by the Vicar, Hugh Farley; the Order prayers were read by George Creech, Knight of St John and the eulogy given by Gavin Proctor.

He is survived by his wife, Olive, whom he married in 1940 and their two children, Judith and Jonathan.

